# Progression of IgM Monoclonal Gammopathy of Renal Significance (MGRS) to Symptomatic Waldenström Macroglobulinemia: A Case Report

**DOI:** 10.1002/cnr2.70281

**Published:** 2025-07-19

**Authors:** Kenichi Ito, Hiroaki Shimoyamada, Kazuhiko Hirano, Naohiro Sekiguchi

**Affiliations:** ^1^ Hematology Division National Hospital Organization Disaster Medical Center Tokyo Japan; ^2^ Pathology Division Dokkyo Medical University Saitama Medical Center Saitama Japan; ^3^ Laboratory and Pathology Division National Hospital Organization Disaster Medical Center Tokyo Japan; ^4^ Clinical Research Division National Hospital Organization Disaster Medical Center Tokyo Japan

**Keywords:** monoclonal gammopathy of renal significance, monoclonal immunoglobulin deposition disease, Tirabrutinib, Waldenstrom macroglobulinemia

## Abstract

**Background:**

Monoclonal gammopathy of renal significance (MGRS) is characterized as renal impairment caused by monoclonal protein but does not fulfill the criteria for specific hematologic malignancies. Most MGRS cases involve IgG, IgA, or light chains, but IgM‐MGRS remains poorly understood.

**Case:**

We present a 74‐year‐old woman with IgM‐κ monoclonal proteinuria who initially declined further evaluation. Later, anemia was identified, and a systemic work‐up revealed monoclonal immunoglobulin deposition disease in the kidney and symptomatic Waldenström macroglobulinemia. Treatment with a Bruton's tyrosine kinase inhibitor, namely tirabrutinib, rapidly resolved both proteinuria and anemia.

**Conclusion:**

This case highlights the importance of early renal biopsy and prompt intervention in suspected IgM‐MGRS.

## 1 Introduction

Monoclonal protein (M‐protein) produced by clonally proliferating B cells and plasma cells can cause organ damage, even in the absence of hematologic malignancies that require therapeutic intervention [1]. When these M‐proteins induce pathological changes in the kidneys without meeting the diagnostic criteria for specific hematologic malignancies, including multiple myeloma (MM) [2] or Waldenström macroglobulinemia (WM) [3], the condition is defined as monoclonal gammopathy of renal significance (MGRS) [4].

MGRS requires therapeutic intervention to prevent progression to severe renal impairment, and delayed intervention can worsen prognosis [5]. Treatment targeting the clones that produce M‐proteins is effective [6, 7, 8], with therapy typically guided by the underlying disease. Specifically, MGRS associated with IgG, IgA, and light chain types is treated similarly to MM, whereas IgM‐MGRS is managed according to the principles of WM, and monoclonal B lymphocytosis is treated based on chronic lymphocytic leukemia protocols [9]. Most reports of MGRS involve IgG and IgA types, but IgM‐MGRS is rare, and consequently, limited reports are available on the pathophysiology and management of IgM‐MGRS [10, 11, 12, 13].

Conversely, WM is defined as lymphoplasmacytic lymphoma with bone marrow involvement and IgM monoclonal protein [3]. A related but distinct entity, IgM monoclonal gammopathy of undetermined significance (MGUS), is characterized by an IgM M‐protein < 3 g/dL, neoplastic cells < 10% in the bone marrow, and no symptoms [14]. When the IgM level ≥ 3 g/dL and/or neoplastic cells ≥ 10% in the bone marrow, the condition is further categorized as either asymptomatic or symptomatic WM, with treatment indicated only for the symptomatic form [15]. In symptomatic WM, nephropathy associated with the disease has also been reported to significantly impact its clinical course, underscoring the importance of effectively managing renal involvement [16].

This case report presents a rare instance of a patient who progressed from IgM‐MGRS to symptomatic WM and was treated with tirabrutinib, resulting in the improvement of renal impairment.

## 2 Case

A 74‐year‐old woman with a history of hypertension and dyslipidemia was referred to the NHO Disaster Medical Center in September 2022 due to proteinuria. Urinalysis revealed urinary protein excretion of 3.0 g/gCr and urinary albumin of 2.0 g/gCr, indicating predominant albuminuria. Urinary sediment showed no apparent red blood cells, but hyaline casts were observed. Laboratory findings included hemoglobin (11.1 g/dL), serum total protein (6.4 g/dL), serum albumin (3.3 g/dL), serum creatinine (0.48 mg/dL), blood urea nitrogen (BUN) (19 mg/dL), Cystatin C (0.94 mg/L) (normal range: 0.47–0.82 mg/L), serum IgG (543 mg/dL) (normal range: 861–1747 mg/dL), serum IgA (69 mg/dL) (normal range: 93–393 mg/dL), serum IgM (984 mg/dL) (normal range: 50–269 mg/dL), kappa light chains (95.7 mg/L) (normal range: 3.3–19.4 mg/dL), and lambda light chains (9.9 mg/L) (normal range: 5.7–16.3 mg/dL). Serum immunofixation electrophoresis detected a monoclonal band of IgM‐κ, and the serum M protein level was 1.16 g/dL. Computed tomography (CT) showed no lymphadenopathy or hepatosplenomegaly. Both kidneys had normal length, but focal cortical thinning was observed. Physical examination revealed no fever, fatigue, anemia‐related symptoms, finger abnormalities, Raynaud's phenomenon, livedo reticularis, or findings suggestive of amyloidosis. Given the IgM M‐protein level was < 3 g/dL and the absence of clinical symptoms, the case was classified as IgM‐MGUS or asymptomatic WM, both of which do not require treatment [15]. However, due to the presence of proteinuria, IgM‐MGRS was suspected [4]; however, the patient declined further evaluation at that time, and therefore, close observation was performed.

In November 2023 (1 year and 2 months after the initial visit), worsening anemia prompted further investigation. Laboratory and urinalysis results included WBC (8100/mm^3^) (67% neutrophils, 21% lymphocytes, 11% monocytes, and 1% eosinophils), hemoglobin (10.1 g/dL), total protein (6.9 g/dL), albumin (3.4 g/dL), BUN (17.5 mg/dL), creatinine (0.48 mg/dL), LDH (130 U/L), serum calcium (9.4 mg/dL), CRP (0.44 mg/dL), IgG (549 mg/dL), IgA (64 mg/dL), IgM (1395 mg/dL), C3 (95 mg/d)L, C4 (29 mg/dL), β2‐microglobulin (2.2 mg/L), kappa light chains (173 mg/L), lambda light chains (10.6 mg/L), urinary protein (4.2 g/gCr), and urinary albumin (2.5 g/gCr). Urinary sediment showed 1–4 red blood cells per high power field, along with the presence of hyaline casts and waxy casts. Serum cryoglobulin was negative. CT imaging showed no apparent lymphadenopathy or splenomegaly.

**FIGURE 1:**
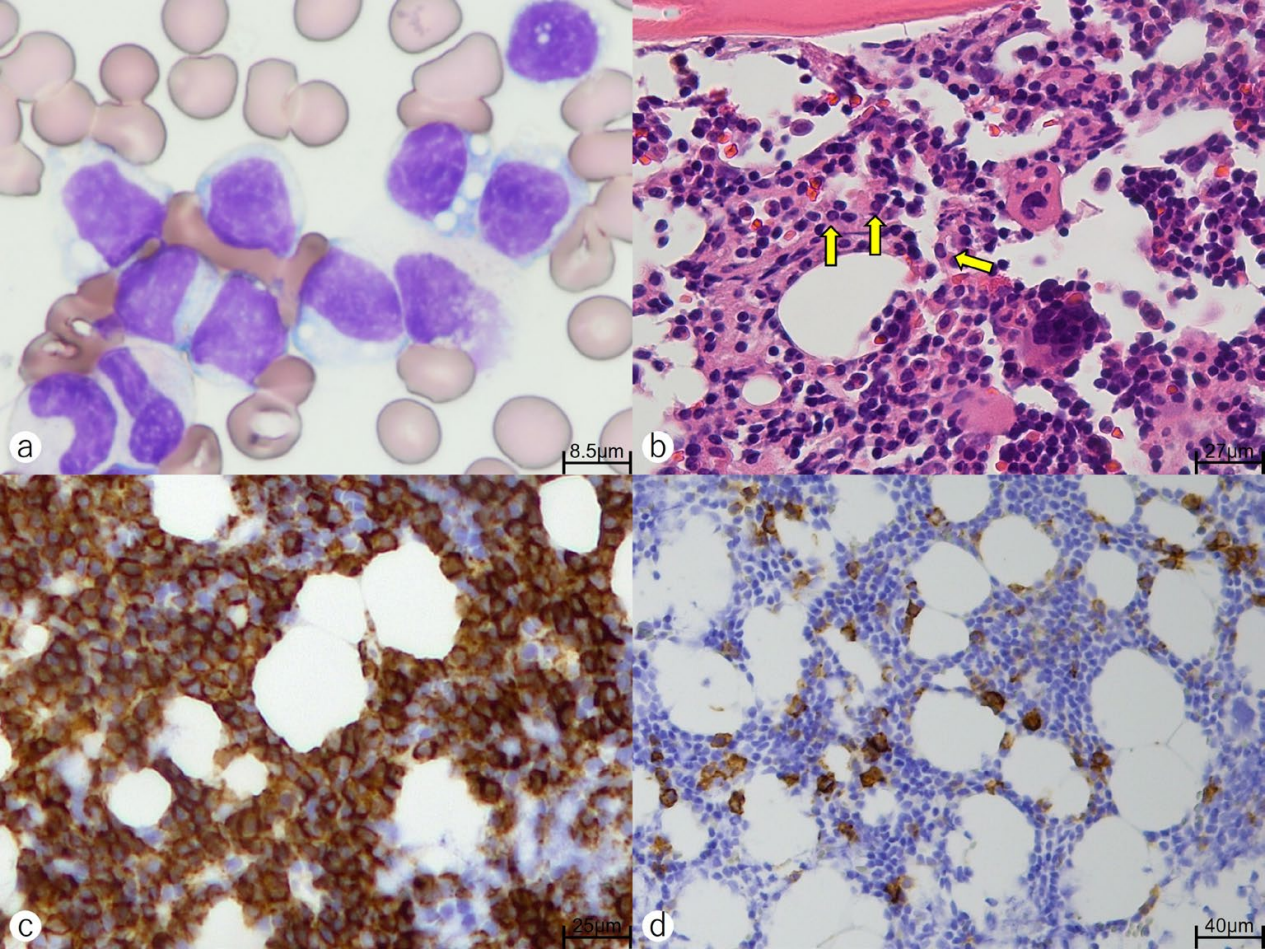
(a) May Grünwald‐Giemsa Stain of a bone marrow specimen. Atypical lymphoplasmacytic cells and small lymphocytes were generally infiltrated (original magnification, 1400×). (b–d) Results of the bone marrow biopsy specimens. (b) Hematoxylin and eosin staining. Diffuse infiltration of neoplastic lymphocytes with plasma cell differentiation in the bone marrow was observed. Plasma cells with a Dutcher body are indicated by yellow arrows (460×). (c) Staining for CD20. Neoplastic lymphocytes were positive for CD20 (500×). (d) Staining for CD138. Neoplastic lymphocytes were partially stained positive for CD138 (300×).

Bone marrow aspirates revealed increased lymphoplasmacytic cells, with approximately 30% tumor burden (Figure 1a). A bone marrow biopsy specimen showed diffuse infiltration of small lymphocytes positive for CD20, as well as plasma cells positive for CD138, IgM, and κ upon immunostaining (Figure 1b–d). Digital droplet PCR detected an *MYD88 L265P* mutation, whereas the *CXCR4 S338X* mutation was absent [17]. G‐banding analysis showed a normal karyotype. Based on these findings, the patient was diagnosed with symptomatic WM [3] and classified as intermediate risk based on the Revised International Prognostic Scoring System for WM [18].

**FIGURE 2:**
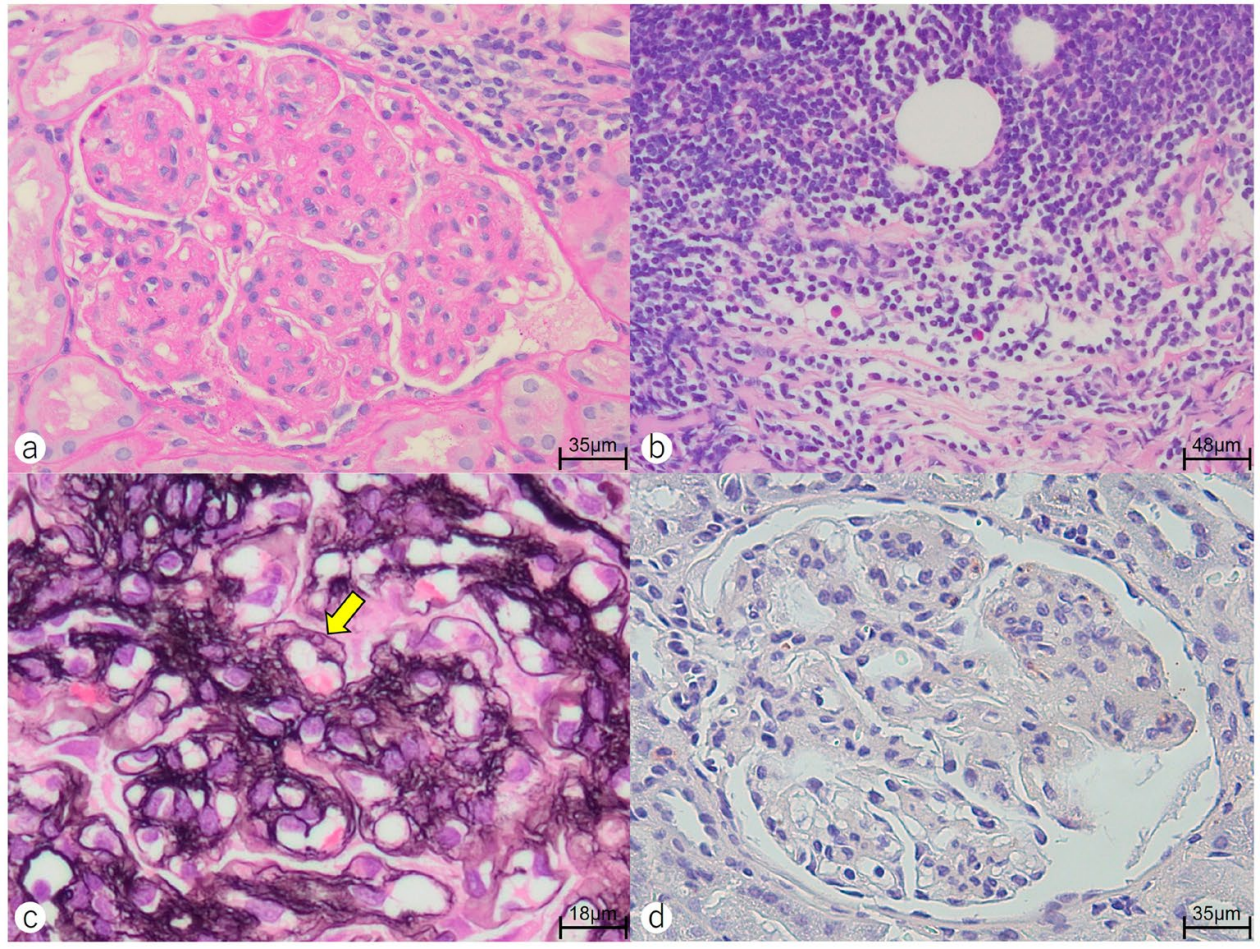
(a, b) Periodic acid‐Schiff staining of the kidney biopsy specimens. (a) A glomerulus with membranoproliferative glomerulonephritis (MPGN)‐like changes, characterized by a lobular architecture resulting from mesangial hypercellularity and matrix expansion (original magnification, 360×). (b) Marked atypical lymphocytes infiltrate at the border between the renal cortex and medulla (260×). (c) Periodic acid‐methenamine silver staining. Extensive double contours of the glomerular basement membranes (indicated with a yellow arrow) were recognized (700×), a characteristic finding of MPGN‐like changes. (d) Dense fine speckled staining, used to detect amyloid, showed no amyloid deposits (360×).

**FIGURE 3:**
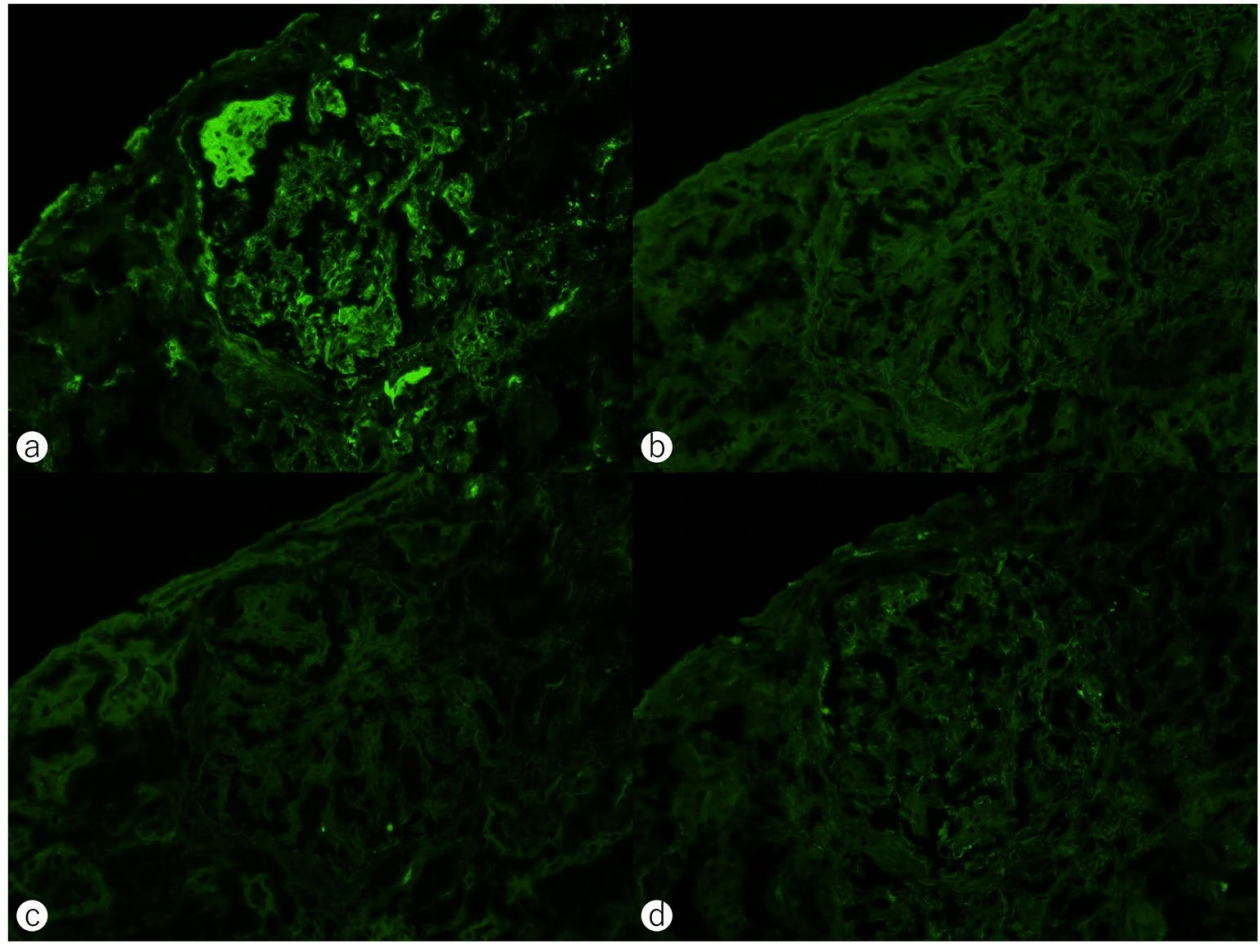
Immunofluorescence microscopy of the kidney biopsy specimens. (a) Immunoglobulin (Ig) M deposits were observed along the glomerular capillary and mesangial lesion. (b–d) No deposition of IgG (b), IgA (c), and C3 (d) was detected.

**FIGURE 4:**
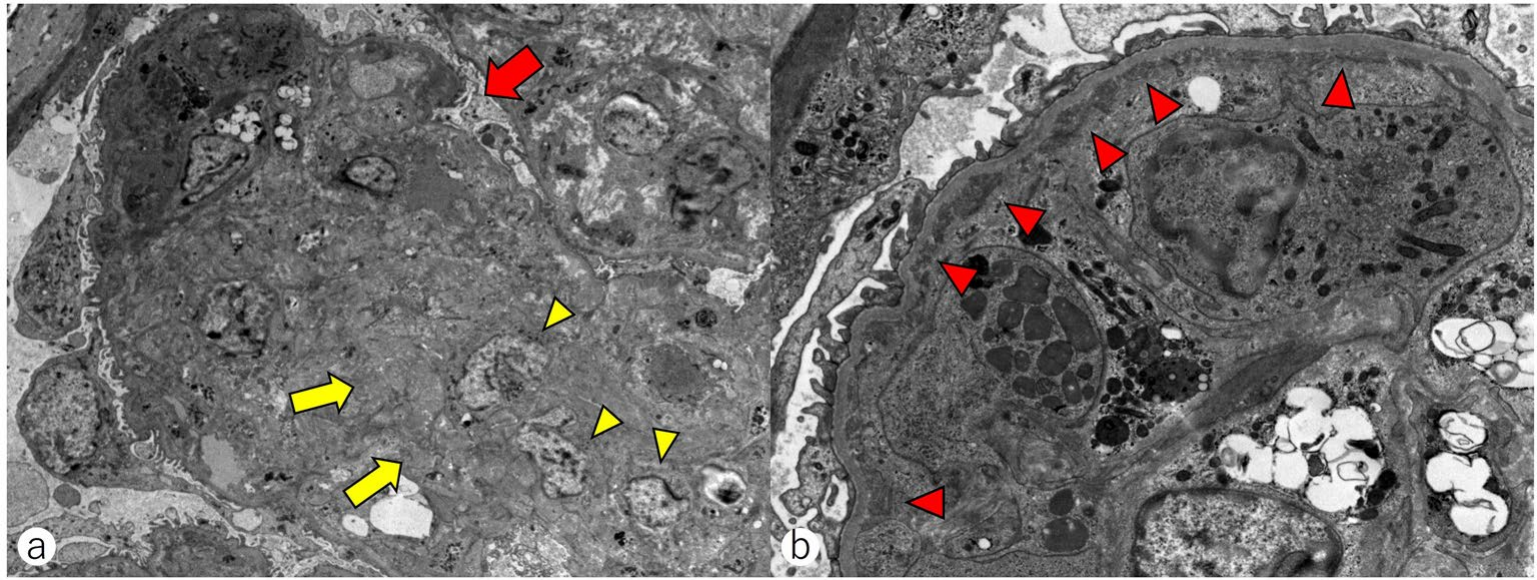
Electron microscopy of the kidney biopsy specimens. (a) Mesangial cell proliferation (yellow arrow heads), matrix expansion (yellow arrows), and lobular glomerular changes (a red arrow) were observed (original magnification, 5000×), consistent with membranoproliferative glomerulonephritis‐like changes. (b) High electron‐dense deposits were identified along the basement membrane (red arrow heads) (25 000×), most likely corresponding to IgM, and consistent with ultrastructural features of IgM‐type proliferative glomerulonephritis with monoclonal IgG deposits.

Periodic acid‐Schiff staining of the kidney biopsy specimen revealed segmental mesangial hypercellularity and matrix expansion with lobular glomerular changes (Figure 2a). Dense infiltration of atypical small lymphocytes was observed in the cortico‐medullary junction (Figure 2b). Glomerular basement membrane duplication was observed by periodic acid‐methenamine silver staining (Figure 2c). No amyloid deposits were detected by dense fine speckled (DFS) staining (Figure 2d). Immunofluorescence staining specimens showed IgM deposition in the mesangium and glomerular capillary loops (Figure 3a), while no deposition of IgG, IgA, and C3 was observed (Figure 3b–d). Electron microscopy revealed mesangial cell proliferation, matrix expansion, and lobular glomerular changes (Figure 4a). High electron‐dense deposits were also observed along the basement membrane (Figure 4b). These findings were consistent with Membranoproliferative glomerulonephritis‐like glomerulonephritis. The renal pathology was attributed to the IgM‐type of proliferative glomerulonephritis with monoclonal IgG deposits (PGNMID) [19] and lymphocytic infiltration. Therefore, at the time of the initial visit, the patient was presumed to have had MGUS or asymptomatic WM with renal involvement—either PGNMID or tumor infiltration—representing IgM‐MGRS. The disease was later diagnosed as progression from IgM‐MGRS to symptomatic WM.

**FIGURE 5:**
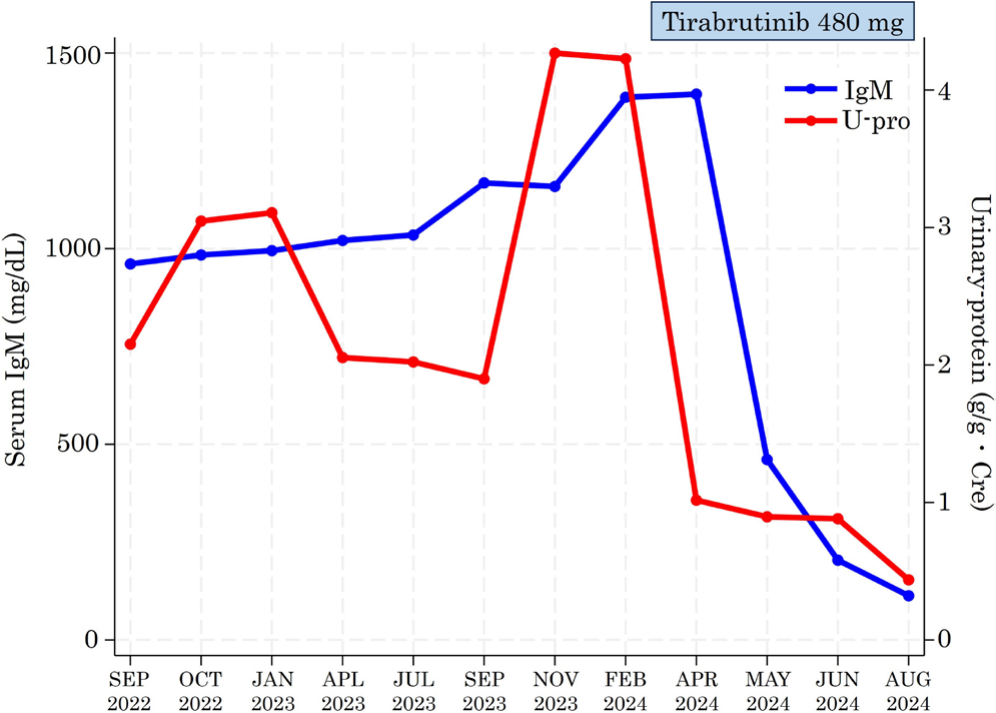
The clinical course of the patient. The blue curve represents the progression of serum Immunoglobulin M levels, and the red curve represents the progression of urinary protein levels. Treatment with tirabrutinib was initiated at the left edge of the blue‐shaded area, after which both IgM and urinary protein levels showed a marked decline.

Currently, Bruton's tyrosine kinase (BTK) inhibitors and chemoimmunotherapy regimens including rituximab are regarded as comparable with respect to major response rates and long‐term survival outcomes for symptomatic WM [20]. However, these modalities differ in terms of route of administration, treatment duration, and adverse event profiles. In Japan, available treatment options include ibrutinib in combination with rituximab, the second‐generation BTK inhibitor tirabrutinib, and conventional chemoimmunotherapy [21]. Following comprehensive shared decision‐making, tirabrutinib was selected as the treatment of choice. Tirabrutinib was orally initiated at a dose of 480 mg once daily [22]. This treatment induced a reduction in serum IgM levels, achieving a partial response, along with a marked decrease in urinary protein excretion (Figure 5). An easily controllable skin rash occurred as an adverse event, yet the therapeutic effect has been sustained for over a year.

## 3 Discussion

In 2012, the International Kidney and Monoclonal Gammopathy Research Group introduced the concept of MGRS, defining it as kidney damage caused by monoclonal proteins requiring therapeutic intervention [23]. Since then, the concept of this disease has gradually become established, although it is still not fully recognized [4, 24]. MGRS arises in the context of MGUS [14, 25]. Despite its asymptomatic nature, MGUS is relatively common; a Japanese population study reported its prevalence at approximately 2.1% [26]. Moreover, MGRS is identified in 1%–1.5% of MGUS cases [27, 28], emphasizing its clinical relevance. Diagnosis of MGRS, however, is challenging because kidney biopsy remains the only definitive diagnostic tool. Given its prevalence and the need for active treatment, early and targeted kidney biopsy is crucial in patients with MGUS who present with renal dysfunction, allowing for the timely identification and management of MGRS.

IgM‐MGRS is a rare condition, comprising approximately 7% of all MGRS cases [12, 29]. Renal lesions associated with IgM‐producing B‐cell neoplasms exhibit diverse clinical manifestations. Higgins et al. analyzed 57 cases of IgM‐producing B‐cell lymphoproliferative disorders (including WM and MGRS) with renal involvement, categorizing renal pathology into amyloid‐related glomerulonephritis (33%), non‐amyloid glomerulonephritis (including cryoglobulinemia and Monoclonal immunoglobulin deposition disease, 35%), tubulointerstitial nephritis (including lymphocytic infiltration and cast nephropathy, 14%), and unrelated renal diseases (18%) [11]. In the present case, amyloidosis was excluded based on negative DFS staining, and cryoglobulinemia was ruled out due to the absence of serum cryoglobulins. Pathological findings confirmed IgM type of PGNMID with lymphocytic infiltration as the underlying cause of MGRS.

IgM MGUS is known to progress to symptomatic WM at an annual rate of 1.5%, approximately 1.5 times higher than the progression rate of non‐IgM MGUS to symptomatic MM [30]. Furthermore, studies have shown that MGRS poses a significantly higher risk of progression to symptomatic MM than MGUS [27]. Herein, the patient progressed to symptomatic WM within 1 year and 5 months, suggesting a potentially high risk of progression in IgM‐MGRS.

Treatment for IgM‐MGRS generally mirrors that of WM, utilizing therapies such as rituximab, bortezomib, and cyclophosphamide [10, 11, 12, 13]. However, no optimal treatment has been established for IgM‐MGRS. Despite the lack of standardized treatment options, early therapeutic intervention is critical. Liu et al. analyzed 38 cases of IgM‐MGRS, reporting that patients who responded to immunochemotherapy had a shorter interval between the onset of renal dysfunction and kidney biopsy (2.9 vs. 6.9 months, *p* = 0.035) [13]. Moreover, patients receiving chemotherapy or autologous stem cell transplantation during the IgM‐MGRS stage had a lower rate of progression to end‐stage renal disease than untreated patients (25% vs. 57%, *p* = 0.049) [13]. These findings underscore the necessity of timely treatment, which was also crucial in the present case.

Regarding WM with renal involvement, the broader impact of renal involvement in WM has been considered. Vos et al. analyzed 1391 WM patients and identified 44 with kidney involvement. They reported that WM patients with renal impairment had significantly shorter overall survival than those without renal impairment (11.5 vs. 16 years, *p* = 0.03) [16]. Furthermore, patients with improved or stable renal function had significantly longer overall survival than those with worsening renal function (*p* = 0.05) [16]. In this case, the use of the BTK inhibitor tirabrutinib in WM following IgM‐MGRS led to rapid improvement in proteinuria and serum monoclonal protein levels. Tirabrutinib's low renal toxicity and its ability to quickly reduce M‐protein levels underscore its potential as a promising treatment option for WM with IgM‐type of PGNMID‐related kidney lesions. These findings may inform medical oncologists/hematologists in selecting effective and renal‐safe therapies for similar cases involving IgM‐related kidney pathology.

## 4 Conclusion

This case report highlights the significant impact of renal involvement in IgM‐MGRS and WM on clinical outcomes, emphasizing the importance of early kidney biopsy and prompt therapeutic intervention. Early biopsy and treatment in this case may have been beneficial. Nonetheless, the favorable clinical response to tirabrutinib—reflected in the rapid improvement of proteinuria and IgM levels—suggests its potential as an effective and renal‐safe treatment option in similar cases. The rarity of IgM‐MGRS and the absence of definitive therapeutic guidelines underscore the need for further clinical studies to establish optimal management strategies. This case may help medical oncologists/hematologists recognize IgM‐MGRS as a diagnostic consideration in patients with unexplained renal impairment and IgM monoclonal gammopathy, and may inform treatment decisions in similar clinical contexts.

## Author Contributions

All authors contributed to the study conception and design. Material preparation, data collection, and analysis were performed by Kenichi Ito, Hiroaki Shimoyamada, and Kazuhiko Hirano. The first draft of the manuscript was written by Kenichi Ito, and all authors commented on previous versions of the manuscript. Naohiro Sekiguchi analyzed the data and revised the manuscript critically for important intellectual content. All authors read and approved the final manuscript.

## Ethics Statement

Because this is a case report, ethical committee approval is not required for this study in accordance with the ethical national guidelines in Japan.

## Consent

Written consent was obtained from the patient for publication of the details of its medical case and any accompanying images.

## Conflicts of Interest

Naohiro Sekiguchi receives honoraria from Janssen, Ono, and research funding from Incyte Biosciences Japan, Janssen, Mitsubishi Tanabe Pharma Corporation, MSD, and Ono.

## Data Availability

The data that support the findings of this study are available on request from the corresponding author. The data are not publicly available due to privacy or ethical restrictions.
